# Supplementary material for: Lipid Nanoparticles Loaded with Farnesol or Geraniol to Enhance the Susceptibility of E. coli MCR-1 to Colistin
Source: Pharmaceutics. 2021 Nov 3;13(11):1849. doi: 10.3390/pharmaceutics13111849 (PMC8625850; doi:10.3390/pharmaceutics13111849)
Supplement: Supplementary file 1 [file pharmaceutics-13-01849-s001.zip › pharmaceutics-1405482-supplementary.pdf]

pharmaceutics MDPI

Supplementary Materials: Lipid Nanoparticles Loaded with Farnesol or Geraniol to Enhance the Susceptibility of *E. coli* MCR-1 to Colistin

Chantal Valcourt, Julien M. Buyck, Nicolas Grégoire, William Couet, Sandrine Marchand, Frédéric Tewes

Table S1. Predicted Emax model parameters describing the variation of **colistin MIC against *E.coli* J53 MCR-1 versus FAR-loaded LNP concentration**. For each parameter, n_eff is a measure of effective sample size and Rhat is the ratios of total variance to within chain variance.

	mean	RSE_mean	sd	2.5%	25%	50%	75%	97.5%	n_eff	Rhat
MIC0	8.68	0.23%	0.56	7.58	8.31	8.67	9.06	9.77	1257	0.9998
ec50	2.15	0.47%	0.24	1.72	1.98	2.14	2.30	2.66	1422	1.0015
gamma	2.14	0.47%	0.35	1.56	1.89	2.11	2.34	2.95	1654	1.0010
sigma	0.45	0.00%	0.10	0.31	0.39	0.44	0.50	0.67	645	1.0025
MIC∞	0.54	0.00%	0.16	0.21	0.44	0.55	0.65	0.86	1436	1.0023

Table S2. Predicted adjustment parameters for Emax model describing the variation of **colistin MIC against *E.coli* J53 versus FAR-loaded LNP** concentration.

	mean	RSE_mean	sd	2.5%	25%	50%	75%	97.5%	n_eff	Rhat
MIC0	0.26	0.00%	0.01	0.23	0.25	0.26	0.27	0.28	2361	1.0010
ec50	1.98	0.51%	0.26	1.50	1.81	1.97	2.14	2.54	2211	1.0010
0gamma	3.10	2.90%	0.28	1.71	2.29	2.65	3.16	8.14	678	1.0010
sigma	0.02	0.00%	0.00	0.01	0.02	0.02	0.02	0.03	787	1.0050
MIC∞	0.06	0.00%	0.00	0.05	0.06	0.06	0.06	0.07	1495	1.0010

Table S3. Predicted adjustment parameters for Emax model describing the variation of **colistin MIC against *E.coli* J53 versus GER-loaded LNP** concentration.

	mean	RSE_mean	sd	2.5%	25%	50%	75%	97.5%	n_eff	Rhat
MIC0	0.26	0.00%	0.01	0.24	0.25	0.26	0.26	0.28	1885	1.0010
ec50	2.00	0.00%	0.21	1.62	1.93	2.03	2.14	2.43	1832	1.0001
gamma	1.93	0.52%	0.28	1.47	1.80	1.93	2.07	2.51	2436	1.0016
sigma	0.02	0.00%	0.00	0.01	0.01	0.02	0.02	0.02	1692	1.0012
MIC∞	0.06	0.00%	0.00	0.05	0.06	0.06	0.06	0.07	2230	1.0003

Table S4. Predicted adjustment parameters for Emax model describing the variation of **colistin MIC against *E.coli J53* MCR-*1* versus GER-loaded LNP** concentration.

	mean	RSE_mean	sd	2.5%	25%	50%	75%	97.5%	n_eff	Rhat
MIC0	8.07	0.32%	0.17	7.75	7.96	8.07	8.18	8.40	2765	1.0010
ec50	35.66	4.59%	2.03	31.96	34.29	35.57	36.91	40.05	1956	1.0011
gamma	4.82	2.64%	1.16	3.00	4.08	4.68	5.40	7.38	1920	1.0006
sigma	0.57	0.30%	0.10	0.41	0.50	0.56	0.63	0.82	1150	1.0063
MIC∞	0.70	0.68%	0.28	0.15	0.50	0.70	0.88	1.27	1727	1.0017

Table S5. Predicted adjustment parameters for **PI uptake in *E.coli J53* induced by colistin**.

	mean	RSE_mean	sd	2.5%	25%	50%	75%	97.5%	n_eff	Rhat
MIC0	0.69	0.00%	0.02	0.65	0.67	0.69	0.7	0.73	2208	1.001
ec50	0.86	0.00%	0.08	0.71	0.8	0.86	0.92	1.02	1281	1.002
gamma	3.48	1.72%	1.62	2.08	2.72	3.14	3.69	7.62	799	1.005
sigma	0.14	0.00%	0.01	0.12	0.13	0.14	0.14	0.15	1687	1.000
MIC∞	0.07	0.00%	0.02	0.03	0.05	0.07	0.08	0.11	1148	1.000

Table S6. Predicted adjustment parameters for **PI uptake in *E.coli J53* MCR-1 induced by colistin**.

	mean	RSE_mean	sd	2.5%	25%	50%	75%	97.5%	n_eff	Rhat
MIC0	0.97	0.00%	0.03	0.89	0.95	0.97	0.99	1	3206	1.0005
ec50	7.38	0.01%	0.85	5.72	6.81	7.4	7.95	9.01	3437	0.9998
gamma	1.03	0.00%	0.1	0.85	0.95	1.02	1.09	1.25	2939	1.0019
sigma	0.15	0.00%	0.01	0.14	0.14	0.15	0.15	0.16	1879	1.0015
MIC∞	0.07	0.00%	0.02	0.03	0.05	0.07	0.08	0.1	3004	1.0011

Table S7. Predicted adjustment parameters for **PI uptake in *E.coli J53* MCR-1** induced by colistin in the presence of **FAR-loaded LNP at 30 mg/L.**

	mean	RSE_mean	sd	2.5%	25%	50%	75%	97.5%	n_eff	Rhat
MIC0	4.25	0.00%	3.97	4.14	4.14	4.24	4.34	4.57	2096	1.0008
ec50	2.69	0.37%	2.24	2.52	2.51	2.67	2.84	3.22	2260	1.0015
gamma	1.56	0.00%	1.24	1.43	1.43	1.55	1.67	2.00	2245	1.0008
sigma	0.29	0.00%	0.23	0.26	0.26	0.28	0.31	0.36	1604	1.0024
MIC∞	0.11	0.00%	0.01	0.06	0.06	0.10	0.15	0.25	2356	1.0009
